# Factors that influence the mortality of patients following hip hemiarthroplasty

**DOI:** 10.1186/s42836-024-00247-1

**Published:** 2024-06-07

**Authors:** Nikit Venishetty, Jonathan Jose, Prabhudev Prasad A. Purudappa, Varatharaj Mounasamy, Senthil Sambandam

**Affiliations:** 1 Paul L. Foster School of Medicine, Texas Tech University Health Sciences Center, El Paso, TX 79905 USA; 2 Carroll Senior High School, Dallas, TX 76092 USA; 3 Boston VA Medical Center, Boston, MA 02130 USA; 4 Department of Orthopedics, University of Texas Southwestern, Dallas VAMC, Dallas, TX 75390 USA

**Keywords:** Hemiarthroplasty, Mortality, Operative complications, Hip fracture

## Abstract

**Introduction:**

Hip hemiarthroplasty (HHA) is one of the most common types of orthopedic surgery. With the prevalence and utilization of the surgery increasing year after year, this procedure is found to be associated with severe postoperative complications and eventually mortality. Thus, it is crucial to understand the factors that increase the risk of mortality following HHA.

**Methods:**

Using the Nationwide Inpatient Sample (NIS) database, patients undergoing HHA from 2016 to 2019 were identified. This sample was stratified into a mortality group and a control group. The data regarding patients’ demographics, co-morbidities, and associated complications were compared between the groups.

**Results:**

Of the 84,067 patients who underwent the HHA procedures, 1,327 (1.6%) patients died. Additionally, the mortality group had a higher percentage of patients who were non-electively admitted (*P* < 0.001) and diabetic patients with complications (*P* < 0.001), but lower incidences of tobacco-related disorders (*P* < 0.001). Significant differences were also seen in age (*P* < 0.001), length of stay (*P* < 0.001), and total charges (*P* < 0.001) between the two groups. Preoperatively, those aged > 70 years (OR: 2.11, 95% CI [1.74, 2.56], *P* < 0.001) had diabetes without complications (OR: 0.32, 95% CI [0.23, 0.44], *P* < 0.001), tobacco-related disorders (OR: 0.24, 95% CI [0.17, 0.34], *P* < 0.001) and increased rates of mortality after HHA. Postoperatively, conditions, such as pulmonary embolisms (OR: 6.62, 95% CI [5.07, 8.65], *P* < 0.001), acute renal failure (OR: 4.58 95% CI [4.09, 5.13], *P* < 0.001), pneumonia (95% CI [2.72, 3.83], *P* < 0.001), and myocardial infarctions (OR: 2.65, 95% CI [1.80, 3.92], *P* < 0.001) increased likelihood of death after undergoing HHA. Patients who were electively admitted (OR: 0.46 95% CI [0.35, 0.61], *P* < 0.001) had preoperative obesity (OR: 0.67, 95% CI [0.44, 0.84], *P* = 0.002), and a periprosthetic dislocation (OR: 0.51, 95% CI [0.31, 0.83], *P* = 0.007) and were found to have a decreased risk of mortality following THA.

**Conclusions:**

Analysis of pre- and postoperative complications relating to HHA revealed that several comorbidities and postoperative complications increased the odds of mortality. Old age, pulmonary embolisms, acute renal failure, pneumonia, and myocardial infraction enhanced the odds of post-HHA mortality.

## Introduction

Hip fractures predispose patients to several complications, reduced quality of life, and an increased risk of subsequent mortality [1–6]. Even though the injury occurs frequently, it is still unclear how to treat displaced femoral neck fractures in senior people. Hip arthroplasty is predicted to grow exponentially in the next twenty years, as counts are predicted to be 1,429,000 by 2040 in the United States alone [7]. Additionally, hemiarthroplasty has become the most common treatment for femoral neck fractures, with over 700,000 cases receiving the surgery annually in the United States by 2050 [7].

Many of these patients are often treated with hip hemiarthroplasty (HHA), a procedure that involves replacing the femoral head with a prosthesis. Shorter surgery time, less blood loss, less technical demands, lighter financial burdens, and a lower dislocation rate are all benefits of HHA [8, 9]. Despite great advances in the postoperative care of THA patients, as this treatment is increasingly used in the elderly population, there will always be a small chance of mortality in these patients [10]. However, the objective of care in the context of an ideal healthcare delivery system would entail identifying the potential risk factors for complications or mortality following HHA, recognizing the factors that can be prevented, and developing strategies to mitigate such adversities to further eliminate any avoidable deaths following elective surgery.

Previous studies have analyzed and discussed the factors that influence mortality after total hip arthroplasty (THA). However, to our knowledge, no studies have assessed the factors that impact post-HHA mortality. The purpose of this study was to query the National Inpatient Sample (NIS) database to analyze the overall prevalence of perioperative risk factors and complications that could potentially lead to early perioperative mortality following HHA.

## Methods

### Study sample

More than seven million hospital stays are documented in the NIS database [11]. Due to the breadth of the sample, it is possible to examine several ethnic groups, including Hispanics, and to provide broad national estimates. Over 49 states, or over 97% of the USA’s population, take part in the Healthcare Cost & Utilization initiative that the NIS evaluates [11]. In addition, the NIS offers critical data on demographics, postoperative problems, and hospitalization features after significant procedures such as RSA. This study is exempt from the approval by Institutional Review Board (IRB) because the de-identified data were made available to the public. All patients with ICD-10, the Tenth Revision, and Clinical Modification/Procedure Coding System (CMP) codes connected to HHA were included in this project using data from 2016 to 2019.

In evaluating demographic variables, age, sex, ethnicity, and the presence of obesity were all taken into account. Additionally discussed were comorbidities, medical problems, and surgical complications. Preoperative variables included tobacco-related disorder, elective admissions, obesity, diabetes with complications, diabetes without complications, Parkinson’s disease, and chronic kidney disease (CKD). Postoperative variables included acute renal failure, dialysis, myocardial infarction, blood loss anemia, pneumonia, pulmonary embolism, deep vein thrombosis (DVT), periprosthetic fracture, periprosthetic dislocation, periprosthetic mechanical complication, periprosthetic infection, superficial surgical site infection (SSI), deep SSI, wound dehiscence, and blood transfusion. We also analyzed the length of stay and cost of care for each cohort. ICD codes utilized in the NIS database served as the only basis for inclusion criteria. Since all variables in the study were based on the ICD codes, no exclusion criteria applied.

### Statistical analysis

Statistical analysis was conducted using SPSS version 27.0 (IBM, Armonk, NY, USA). The *t*-test and the Chi-square test were used, respectively, to assess numerical and categorical variables. For variables with an incidence of fewer than 5, Fischer’s exact test was employed. Multivariate analysis (MVA) was then carried out on the factors that had shown a significant correlation with univariate analysis (UVA).

## Results

### Analysis of patient demographics

A total of 84,067 patients were identified in the NIS database during the study period (Table 1). Of these patients, 1,327 (1.6%) died after HHA while the remaining 82,740 were part of the control group. These demographic data can be found in Table 1. With respect to the gender difference, females made up a significant proportion of the mortality (56.28%) of the mortality group and the control group (67.28%), compared to males (*P* < 0.001). Statistically significant differences were found in the rate of obesity between the control and mortality groups (6.29% vs. 3.47%, *P* < 0.001). No statistically significant differences in ethnicity were found between the mortality and control groups. Despite this, Caucasians had a significantly higher proportion in the mortality and control groups compared to African-Americans, Hispanics, Asians, and Native Americans.

**Table 1:** Demographic characteristics in the mortality group and the control group

	**Mortality group** (*n* = 1,327)	**Control group** (*n* = 82,740)	**Significance**
Female	746 (56.28%)	55,666 (67.28%)	** < 0.001**
Minority	144 (10.85%)	9,530 (11.52%)	** < 0.001**
Race			** < 0.001**
Caucasian	1,115 (84.02%)	69,418 (83.90%)	** < 0.001**
African American	74 (5.58%)	3,879 (4.69%)	** < 0.001**
Hispanic	49 (3.69%)	3,911 (4.73%)	** < 0.001**
Asian	17 (1.28%)	1,406 (1.70%)	** < 0.001**
Native American	(0.30%)^a^	334 (0.40%)	** < 0.001**

Significance with *P* < 0.05 was shown in bold type

^a^Numbers between 1 and 10 were not reported per the healthcare cost and utilization project data agreement

### Univariate analysis of mortality and control groups

The number of tobacco-related disorders was much higher in the control group than in the mortality group (Mortality: 2.41%, Control: 8.91% *P* < 0.001). Control group patients were also more likely to be electively admitted than their mortality group counterparts (Mortality: 4.45%, Control: 10.39%, *P* < 0.001). While there was no significant difference in patients who had diabetes with complications between the two groups, a significant difference was found between the two groups in diabetic patients without complications (Mortality: 8.79%, Control: 2.86%, *P* < 0.001) (Table 2). The HHA mortality group had a significantly higher rate of acute renal failure (Mortality: 45.59%, Control: 13.97%, *P* < 0.001), myocardial infarction (Mortality: 2.34%, Control: 0.47%, *P* < 0.001), pneumonia (Mortality: 12.96%, Control: 3.19%, *P* < 0.001), pulmonary embolism (Mortality: 5.95%, Control: 0.68%, *P* < 0.001), DVT (Mortality: 2.34%, Control: 0.80%, *P* < 0.001), CKD (Mortality: 18.01%, Control: 15.15%, *P* = 0.002), blood transfusion (Mortality: 14.85%, Control: 11.67%, *P* < 0.001), and wound dehiscence (Mortality: 0.98%, Control: 0.31%, *P* < 0.001) (Table 3). The HHA control group had a significantly higher rate of Parkinson’s disease (Mortality: 2.19%, Control: 3.94%, *P* < 0.001), periprosthetic Dislocation (Mortality: 1.21%, Control: 2.90%, *P* < 0.001), and periprosthetic mechanical complication (Mortality: 0.00%, Control: 0.86%, *P* < 0.001). No significant differences were found between patients in the mortality and control group who underwent dialysis or had HIV, blood loss anemia, periprosthetic fracture, periprosthetic infection, or SSI. Finally, significant differences were found in age (years) (Mortality: 82.89, Control: 79.24, *P* < 0.001), length of stay (days) (Mortality: 8.30, Control: 5.61, *P* < 0.001), and total charges (in dollars) (Mortality: 139,370.37, Control: 82,539.16, *P* < 0.001) (Table 4).

**Table 2:** Preoperative univariate analysis on patients in HHA mortality and the control group

**Variable**	**Mortality group** (*n* = 1,327)	**Control group** (*n* = 82,740)	**Significance**
Tobacco-related disorder	32 (2.41%)	7,370 (8.91%)	** < 0.001**
Elective admission	59 (4.45%)	8,600 (10.39%)	** < 0.001**
Obesity	46 (3.47%)	5,204 (6.29%)	** < 0.001**
Diabetes without complications	38 (2.86%)	7,269 (8.79%)	** < 0.001**
Diabetes with complications	(0.08%)^a^	206 (0.25%)	0.16
Parkinson’s disease	29 (2.19%)	3,261 (3.94%)	** < 0.001**
Chronic Kidney Disease (CKD)	239 (18.01%)	12,539 (15.15%)	0.002

Significance with* P* < 0.05 was shown in bold type

^a^Numbers between 1 and 10 were not reported per the healthcare cost and utilization project data agreement

**Table 3:** Postoperative univariate analysis on patients in HHA mortality and the control group

**Variable**	**Mortality group** (*n* = 1,327)	**Control group** (*n* = 82,740)	**Significance**
Acute renal failure	605 (45.59%)	11,560 (13.97%)	** < 0.001**
Dialysis	(0.68%)^a^	521 (0.63%)	0.46
Myocardial infarction	31 (2.34%)	388 (0.47%)	** < 0.001**
HIV	(0.23%)^a^	89 (0.11%)	0.18
Blood loss anemia	404 (30.44%)	26,816 (32.41%)	0.068
Pneumonia	172 (12.96%)	2,643 (3.19%)	** < 0.001**
Pulmonary embolism	79 (5.95%)	562 (0.68%)	** < 0.001**
DVT	31 (2.34%)	666 (0.80%)	** < 0.001**
Periprosthetic fracture	40 (3.01%)	2,828 (3.42%)	0.234
Periprosthetic dislocation	16 (1.21%)	2,402 (2.90%)	** < 0.001**
Periprosthetic mechanical complication	(0.00%)^a^	712 (0.86%)	** < 0.001**
Periprosthetic infection	26 (1.96%)	1,674 (2.02%)	0.474
Superficial SSI	(0.08%)^a^	43 (0.05%)	0.504
Deep SSI	(0.00%)^a^	21 (0.03%)	0.716
Wound dehiscence	13 (0.98%)	260 (0.31%)	** < 0.001**
Blood transfusion	197 (14.85%)	9,656 (11.67%)	** < 0.001**

Significance with* P* < 0.05 was shown in bold type

*DVT* deep vein thrombosis, *SSI* surgical site infection, *HIV* human immunodeficiency virus infection

^a^Numbers between 1 and 10 were not reported per the healthcare cost and utilization project data agreement

**Table 4:** Univariate analysis HHA mortality group patients and the control group patients

Variable	Mortality group	Control group	Significance
Age (years)	82.89 (SD = 8.45)	79.24 (SD = 10.30)	** < 0.001**
Length of stay	8.30 (SD = 8.66)	5.61 (SD = 4.513)	** < 0.001**
Total charges	139,370.07 (SD = 183,496.50)	82,539.16 (SD = 63,810.132)	** < 0.001**

Significance with* P* < 0.05 was shown in bold type

### Preoperative multivariate analysis

Multivariate analysis, as utilized in this study, is briefly defined as a statistical technique that evaluates at least 2 variables and attempts to find a possible association between them. Accordingly, many preoperative conditions were found to be correlated with a higher likelihood of death from the HHA procedure (Table 5). Patients aged 70 and older were 2.11 times more likely to die after HHA than younger patients (95% CI [1.74, 2.56], *P* < 0.001). However, diabetic patients without complications (OR: 0.32, 95% CI [0.23, 0.44], *P* < 0.001) and those with tobacco-related disorders (OR: 0.24, 95% CI [0.17, 0.34], *P* < 0.001) were significantly less likely to die after HHA. Females were also less likely to die after HHA (OR: 0.56, 95% CI [0.50, 0.63], *P* < 0.001) as compared to males. Those who had Parkinson’s (OR: 0.4, 95% CI [0.27, 0.60], *P* < 0.001) or suffered from obesity (OR: 0.67, 95% CI [0.44, 0.84], *P* = 0.002) also had decreased likelihood of mortality following HHA. Finally, those who underwent elective HHA were 0.46 times more likely to die than those who received non-elective HHA (95% CI [0.35, 0.61], *P* < 0.001).

**Table 5:** Preoperative multivariate analysis on patients in HHA mortality and the control group

Variable	Odds ratio	Odds ratio 95% CI	Significance
Elective admission	0.46	(0.35, 0.61)	** < 0.001**
Female	0.56	(0.50, 0.63)	** < 0.001**
Diabetes without complications	0.32	(0.23, 0.44)	** < 0.001**
Tobacco related disorder	0.24	(0.17, 0.34)	** < 0.001**
Chronic Kidney Disease (CKD)	0.98	(0.85, 1.14)	0.834
Parkinson’s disease	0.40	(0.27, 0.60)	** < 0.001**
Obesity	0.61	(0.44, 0.84)	**0.002**
Age of 70 and above	2.11	(1.74, 2.56)	** < 0.001**

Significance with* P* < 0.05 was shown in bold type

### Postoperative multivariate analysis

Numerous postoperative conditions resulted in a higher likelihood of death from the HHA (Table 6). Those who had a pulmonary embolism were 6.62 times more likely to die from HHA than those without it (95% CI [5.07, 8.65], *P* < 0.001). Patients with acute renal failure (95% CI [4.09, 5.13], *P* < 0.001) were 4.58 times more likely to die than those without the condition. Additionally, patients with other conditions such as pneumonia (OR: 3.22, 95% CI [2.72, 3.83], *P* < 0.001) and myocardial infarction (OR: 2.65, 95% CI [1.80, 3.92], *P* < 0.001) had also significantly increased likelihood of death compared to those without it. Wound dehiscence patients were 2.92 times more likely to die after HHA than those without the complication (95% CI [1.64, 5.20], *P* < 0.001). However, the only postoperative condition with a lower likelihood of death from HHA was a periprosthetic dislocation (OR: 0.51, 95% CI [0.31, 0.83], *P* = 0.007). Additionally, when the preoperative comorbidities and postoperative complications were matched, numerous associations were found. Conditions such as acute renal failure (OR: 4.26, 95% CI [3.55, 5.11], *P* < 0.001), myocardial infarction (OR: 4.34, 95% CI [1.90, 9.89], *P* < 0.001), pneumonia (OR: 3.73 95% CI [2.69, 5.18], *P* < 0.001), pulmonary embolism (OR: 8.92, 95% CI [4.46, 17.85], *P* < 0.001), DVT (OR: 3.37, 95% CI [1.60, 7.11], *P* < 0.001), and wound dehiscence (OR: 12.62, 95% CI [1.65, 96.64], *P* < 0.001) all displayed increased likelihood of mortality after HHA. However, a periprosthetic dislocation (OR: 0.45, 95% CI [0.25, 0.81], *P* = 0.009) was correlated with a decreased likelihood of mortality after the HHA by greater than a factor of two (Table 7).

**Table 6:** Postoperative multivariate analysis on patients in HHA mortality and control group

Variable	Odds ratio	Odds ratio 95% CI	Significance
Acute renal failure	4.58	(4.09, 5.13)	** < 0.001**
Myocardial Infarction	2.65	(1.80, 3.92)	** < 0.001**
Pneumonia	3.22	(2.72, 3.83)	** < 0.001**
Pulmonary embolism	6.62	(5.07, 8.65)	** < 0.001**
Deep Vein Thrombosis (DVT)	1.14	(0.76, 1.71)	0.528
Periprosthetic dislocation	0.51	(0.31, 0.83)	**0.007**
Periprosthetic mechanical complication	0.00	-	0.991
Wound dehiscence	2.92	(1.64, 5.20)	** < 0.001**
Blood transfusion	1.00	(0.86, 1.17)	0.989

Significance for* P* < 0.05 was shown with bold type

**Table 7:** Multivariate analysis on matched patients in mortality and control groups, with preoperative and postoperative variables combined

Variable	Odds ratio	Odds ratio 95% CI	Significance
Acute renal failure	4.26	(3.55, 5.11)	** < 0.001**
Myocardial infarction	4.34	(1.90, 9.89)	** < 0.001**
Blood loss anemia	0.91	(0.77, 1.07)	0.272
Pneumonia	3.73	(2.69, 5.18)	** < 0.001**
Pulmonary embolism	8.92	(4.46, 17.85)	** < 0.001**
DVT	3.37	(1.60, 7.11)	** < 0.001**
Periprosthetic fracture	0.91	(0.59, 1.42)	0.737
Periprosthetic dislocation	0.45	(0.25, 0.81)	**0.009**
Periprosthetic mechanical complication	0.49	(0.47, 0.51)	0.058
Periprosthetic infection	1.26	(0.70, 2.26)	0.461
Superficial SSI	0.48	(0.04, 5.31)	0.618
Deep SSI	-	-	-
Wound dehiscence	12.62	(1.65, 96.64)	**0.002**
Blood transfusion	1.24	(0.99, 1.55)	0.068

Significance with *P* < 0.05 was shown in bold type

*DVT* deep vein thrombosis, *SSI* surgical site infection

## Discussion

Acute renal failure (ARF) increased the likelihood of mortality in patients who underwent HHA (Table 6, Row 1). Previous studies have reported that acute renal failure in patients undergoing total hip arthroplasty led to higher mortality rates [12, 13]. Hypovolemia resulting in pre-renal kidney injury has been linked to higher rates of mortality [14]. In addition, nephrotoxic drugs used in procedures and clinical settings have also been reported to contribute to worsening ARF and subsequent mortality, potentially due to the drug’s ability to alter the renal structure and function after the surgery [14–16].

Another factor that caused mortality to rise after HHA was myocardial infarction (MI) (Table 6, Row 2). Previous studies have found that patients aged > 85 years receiving hemiarthroplasties carried a significantly higher risk of myocardial infarctions, which, in turn, led to associated mortality risks [1]. Following hip fracture repair, postoperative MI was associated with increased one-year mortality [17]. Additionally, higher troponin levels were associated with the development of MI and MCC (major cardiac complications) [2, 18, 19]. While further research on this relationship is needed, it is clear that cardiac troponin can be utilized as an indicator for the onset of cardiac events that result in higher mortality after procedures such as HHA [20]. In clinical environments, the study concluded that troponin has been assessed to be a useful and reliable biomarker in indicating cardiac complications.

Moreover, we found that patients with pneumonia had higher odds of mortality (Table 6, Row 3). Previous studies analyzed risk factors that influence survivorship after the procedure showed that hemiarthroplasty resulted in significantly higher mortality rates [21]. A few factors might explain this. First, low serum albumin, which is prevalent in hip fracture patients, has been found to be a significant risk factor for the development of postoperative pneumonia (POP) [22, 23]. In addition, those who have undergone the HHA have also been shown to be at a higher risk of infection of the lower respiratory tract, leading to the deterioration of pneumonia and subsequent death [1].

In addition, pulmonary embolism increased the likelihood of mortality in HHA patients, the finding being similar to previous results (Table 6, Row 4) [3, 24]. The study by Shah et al. concludes that hospitals with low volumes were associated with an increased risk for pulmonary embolism after HHA [3]. Specific clinical procedures, such as cemented HA, also led to a statistically significant increase in the rate of PE [24]. This can be explained by the increase in intramedullary pressure during cemented HHA, which may cause a pulmonary embolism due to fat, air, and bone marrow go into the lung, causing a lack of blood flow that can lead to lung tissue damage and subsequent death [25, 26]. Additionally, the type of approach could affect this; a study found that pulmonary embolism was more common after the posterior approach of HHA [27]. Therefore, it is critical to track essential vital signs during surgery to assess the probability of the onset of these complications. It is also extremely valuable to note which approach to the surgery best suits the patient profile, as some approaches of HHA lead to greater instances of certain complications than others.

Contrary to prevailing literature, periprosthetic dislocation appears to reduce mortality after the HHA procedure (Table 6, Row 6). It’s notable that dislocations are prevalent among patients who have undergone hemiarthroplasty for femoral neck fractures [28]. This is significant, as patients with these dislocations have a higher risk of mortality than those without them [29]. Several factors could explain this correlation. For instance, recurrent dislocations can heighten the mortality risk by almost 1.5 times within 90 days post-operation [29]. Additionally, a delay in surgery and closed reduction were also key factors that increased dislocations and subsequent mortality [30]. Moreover, research indicated that patients with an acetabular depth of ≤ 19.12 mm and a corresponding depth-width ratio are especially susceptible to dislocations, heightening their risk of death [4]. Finally, another factor that increased the likelihood of mortality after HHA was wound dehiscence (Table 6, Row 8). Prior studies displayed that HHA carries a higher risk of developing deep infections, which slightly increased mortality rates [31, 32].

Interestingly, our multivariate analysis showed that obesity decreased the odds of mortality (Table 5, Row 7). Previous studies have found an inverse relationship between body weight and mortality following hip fracture surgeries [28]. This so-called “obesity paradox” has been studied and many studies have demonstrated that obesity can be protective against specific diseases such as stroke [33]. Possible explanations for this phenomenon state that the adipose tissue reserves serve as an energy reservoir, resulting in more favorable neuroendocrine profiles to satisfy the raised metabolic needs following surgery [33].

On the other hand, we found that elderly patients (> 70 years of age) also had increased odds of mortality after HHA (Table 5, Row 8). Elderly patients have higher rates of malnutrition and frailty and have been demonstrated to have a higher mortality after hip fracture [34]. These patients usually have lower functional capacity and worse recovery after hip fracture repairs, which may explain their worse outcomes after HHA [34]. When further assessed the demographic variables, and found that female gender was also associated with decreased odds of mortality after HHA, similar to previous studies (Table 5, Row 2) [35, 36]. Moreover, contrary to previous studies, our results exhibited that diabetes and tobacco-related disorders decreased the odds of mortality after HHA (Table 5, Rows 3 and 4) [37, 38]. Lastly, we found that patients with Parkinson’s disease also had decreased mortality following HHA (Table 5, Row 6). While there are studies that suggested the opposite, some studies reported no difference in recovery and mortality outcomes after hip fractures in these patients [39].

## Limitations

Our study has several limitations. Our analysis was based on historical data from the NIS database registry. The records of the NIS database on postoperative adverse occurrences are restricted to information obtained during their hospital stay. The database is, to a great extent, dependent on the availability of accurate diagnosis coding and documentation or techniques. Since the data are a major component of our study, some of the information on co-morbidities and perioperative complications in the NIS database, may probably have been missed. In addition, long-term outcomes regarding patient care could not be assessed, as the NIS only provides data regarding in-hospital adverse events. In addition, we were unable to obtain data regarding relevant factors, including the type of anesthesia used preoperatively, surgical approach, and types of implants being used. Moreover, the time and cause of death for these patients were not available in the NIS database, which might impact the reliability of the results. However, the large sample size may provide care-givers with key information conducive to the care of HHA patients in the perioperative setting.

## Conclusions

Our analysis of the NIS database provided key information on the utilization of HHA. Overall, we found that it is a safe surgery with a low mortality rate during the early postoperative stage. Among various factors analyzed, we found that age was significantly associated with increased odds of mortality. Moreover, factors such as diabetes, female gender, and obesity decreased the odds of mortality after HHA. Additionally, postoperative conditions, including pulmonary embolism, acute renal failure, pneumonia, and myocardial infarction, significantly increased the odds of post-HHA mortality. Our findings suggest that to reduce the overall post-HHA mortality rates, routine testing for the aforementioned comorbidities and risk factors as well as adequate preoperative patient optimization may be advised.

## Data Availability

The datasets used and/or analyzed during the current study are available from the corresponding author on reasonable request.
